# 2D Palladium Sulphate for Visible‐Light‐Driven Optoelectronic Reversible Gas Sensing at Room Temperature

**DOI:** 10.1002/smsc.202100097

**Published:** 2021-12-08

**Authors:** Turki Alkathiri, Kai Xu, Bao Yue Zhang, Muhammad Waqas Khan, Azmira Jannat, Nitu Syed, Ahmed F. M. Almutairi, Nam Ha, Manal M. Y. A. Alsaif, Naresha Pillai, Zhong Li, Torben Daeneke, Jian Zhen Ou

**Affiliations:** ^1^ School of Engineering RMIT University Melbourne 3001 Australia; ^2^ School of Engineering Albaha University Albaha 65779 Saudi Arabia; ^3^ Department of Electrical Engineering Kuwait University Safat 13060 Kuwait; ^4^ Key Laboratory of Advanced Technologies of Materials School of Materials Science and Engineering Southwest Jiaotong University Chengdu 610031 China

**Keywords:** 2D PdSO_4_ nanosheets, narrow bandgap, optoelectronic gas sensing, prolonged exciton lifetime

## Abstract

2D metal sulphides (MSs) have attracted enormous amounts of attention in developing high‐performance gas sensors. 2D noble metal sulphides and their derivatives, however, have been less studied due to their predominant nonlayered crystal structures for inefficient exfoliation, despite their surface and peculiar optoelectronic properties. Herein, we successfully synthesize 2D palladium sulphate (PdSO_4_) from palladium sulphide (PdS) bulk crystals by liquid‐phase exfoliation, in which the presence of oxygen species in the exfoliation solvent plays a key role in the sulphate transformation. Ultrathin 2D PdSO_4_ planar nanosheets, with thicknesses of ≈3 nm and submicrometer lateral dimensions, exhibit a broad absorption across the visible spectrum, a narrow bandgap of ≈1.35 eV, and a nanosecond scaled long exciton lifetime, which are all suitable for the visible‐light‐driven optoelectronic gas sensing applications. The 2D PdSO_4_‐based sensor demonstrates a reversible, selective, and sensitive response toward ppb‐leveled NO_2_ gas at blue light irradiation, featuring a response factor of ≈3.28% for 160 ppb NO_2_, a low limit of detection of 1.84 ppb, and *a* > 3 times response factor enhancement over other gases. Herein, the possibility of realizing 2D ultrathin noble metal sulphide compounds from their nonlayered crystal structures and strong potentials in developing high‐performance chemical sensors is explored.

## Graphical abstract

2D palladium sulphate (PdSO_4_) nanosheets have successfully been delaminated from palladium sulphide bulk crystals utilizing liquid‐phase exfoliation featuring sub‐micron lateral dimensions, nano‐scale thicknesses, and a narrow bandgap. Upon blue light irradiation, 2D PdSO_4_‐based sensor exhibits reversible, selective, and sensitive responses toward nitrogen dioxide (NO_2_) gas with a response factor of 3.28% for 160 parts per billion (ppb).

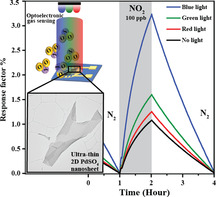

## 1 Introduction

2D atomically thin materials have been explored in various categories of materials ranging from insulators, semiconductors, semimetals, and metals, showing distinctly different properties compared with those of bulk systems.^[^ 1, 2, 3, 4 ^]^ Metal sulphides (MSs) are one of the most studied groups due to their wide implementation in numerous potential applications including optoelectronic devices, energy storage, conversion applications, solar cell, and sensors.^[^ 5, 6, 7, 8 ^]^ Compared with graphene and hexagonal boron nitride (h‐BN) which have a zero and wide bandgap, respectively, MSs have a sizable bandgap and therefore exhibit diverse optical and electronic properties associated with their thicknesses.^[^ 9, 10, 11, 12 ^]^ Palladium sulphide (PdS) is a noble metal compound with bandgap energies between 1.5 and 2 eV, making it a promising candidate in photonics and optical sensing applications originating from their intrinsic optical properties particularly the surface plasmon resonance.^[^ 13, 14, 15 ^]^ However, it has attracted less attention in its 2D planar form owing to its nonlayered crystal feature. Up to date, 0D particle form has been extensively achieved for PdS, mostly involving a mixture of at least two elements under high calcination temperatures.^[^ 16, 17 ^]^ Therefore, the realization of PdS‐based 2D materials, preferably utilizing inexpensive and straightforward approaches, can be beneficial to fully explore its potentials in optoelectronic and sensing applications.

Liquid phase exfoliation has been considering a powerful technique to obtain large quantities of 2D metal sulphides from the mechanical exfoliation of the bulk even with the nonlayered crystal structures using less hazardous chemicals in an ambient environment.^[^ 18, 19, 20, 21 ^]^ Nonlayered materials (i.e., isotropic materials) have strong atom covalent bonds in all three dimensions, preventing them to be easily exfoliated utilizing conventional techniques. However, some nonlayered materials, such as group‐10 metal mono/dichalcogenides (MX_2−*y*_; M: Pd, Pt, etc. and X: S, Se, and Te; and *y* = 1), show excellent anisotropy properties similar to that of graphene, allowing them to be exfoliated into layer‐like structures.^[^ 22, 23 ^]^ The weak adjacent bonds forces in these materials can be broken by applying an external force with the aid of a proper solvent.^[^ 24, 25 ^]^ This phenomenon also gives opportunities to explore other 2D non‐layered nanomaterials. Recently, 2D ultrathin nonlayered Te and Se nanosheets are successfully exfoliated from their bulk counterpart through liquid‐phase exfoliation, featuring small lateral dimensions and unique optical properties.^[^ 25, 26 ^]^

Herein, we synthesize 2D planar palladium sulphate (PdSO_4_) from the nonlayered bulk PdS microsized powder using an optimized liquid exfoliation route for the first time, taking the advantages of surface modification and crystal renucleation by the strong localized mechanical force in the liquid medium. In the presence of the strong mechanical force, the bulk PdS is exfoliated into the 2D form with rich surface dangling bonds and sulphur vacancy sites. With the abundant oxygen species dissolved in dimethylformamide (DMF) solvent and the localized evaluated temperatures, those surface defects on 2D PdS crystal can be further oxidized due to the oxygen diffusion, facilitating the formation of the oxysulphide/sulphate phase. Such an liquid phase exfoliation (LPE)‐induced surface nucleation process has also been previously observed in several metal chalcogenides.^[^ 21, 27, 28, 29, 30 ^]^ Considering that 2D metal chalcogenides and oxychalcogenides are promising candidates for room temperature gas sensing applications.^[^ 31, 32, 33, 34, 35 ^]^ The obtained 2D PdSO_4_ nanosheets with submicro lateral dimensions and average thickness of 3 nm are further incorporated into a chemiresistive transducing platform to perform NO_2_ gas sensing at room temperature under light illuminating conditions. 2D PdSO_4_ sensor demonstrates a reversible, sensitive, and selective NO_2_ sensing performance under low‐power light irradiation at room temperature, mainly ascribed to the intrinsically strong light absorption behaviour at visible‐light‐spectrum, unique gas adsorption manner, as well as the prolonged exciton lifetime of 2D PdSO_4_.

## 2 Results and Discussion

**Figure 1:**
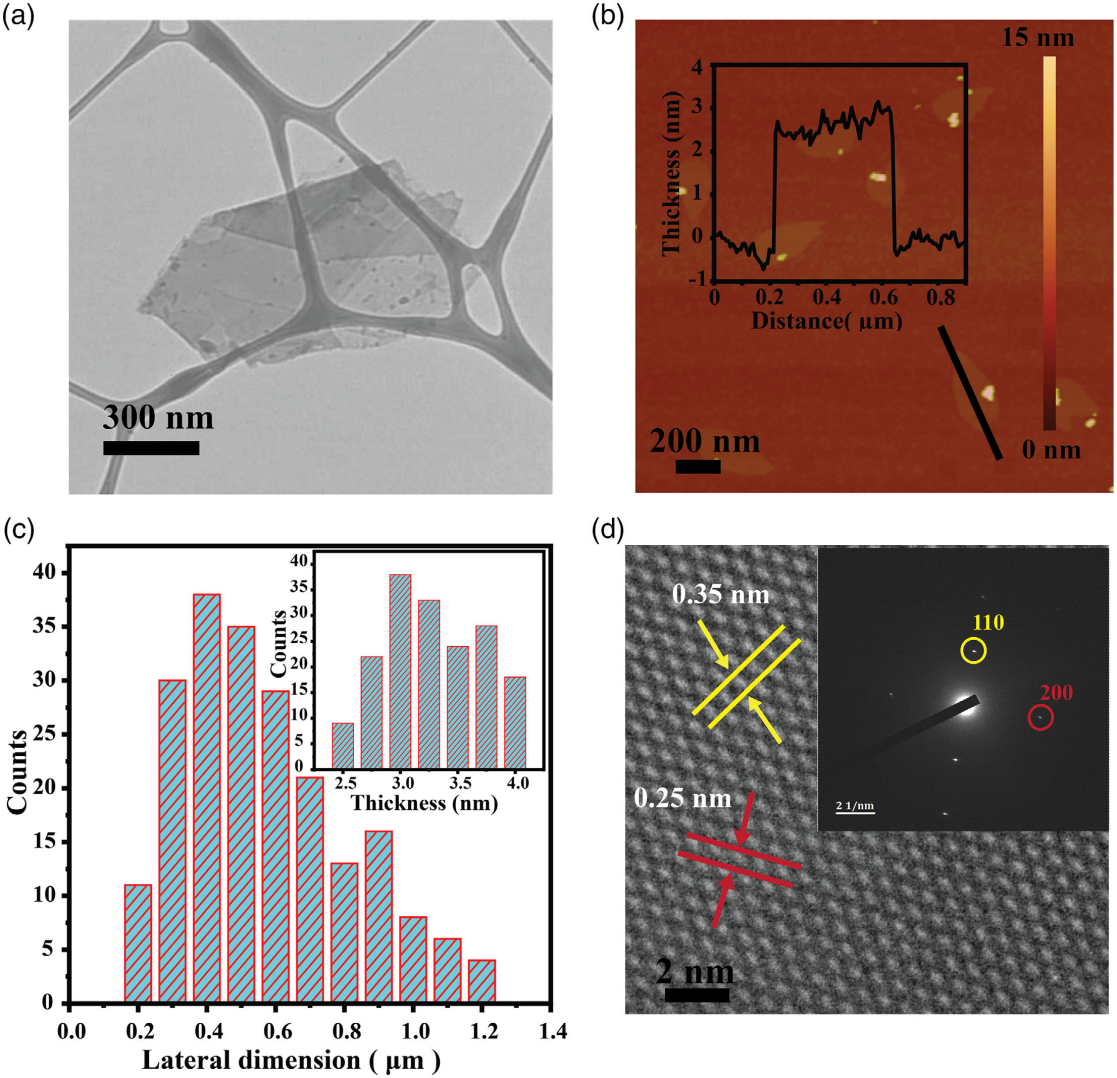
Morphological characterizations: a) typical TEM characterization of 2D PdSO_4_ nanosheets. b) AFM image of the produced nanosheet with its respective thickness profile of ≈3 nm. c) Lateral and thickness distribution were collected from 266 nanosheets. d) HRTEM image indicates the observed lattices spacing 0.35 and 0.25 nm corresponding to planes 110 and 200, respectively, and the inset represents the SAED pattern of the corresponding transmission electron microscope.

The structure and morphology of the exfoliated 2D nanosheets were characterized using transmission electron microscopy (TEM) and atomic force microscope (AFM). A representative low‐magniﬁcation TEM image reveals that the bulk PdS powder has been successfully synthesized into the translucent 2D form with a lateral dimension of ≈0.9 μm (**Figure** **1**a). As shown in the AFM image of a typical nanosheet Figure 1b, the obtained 2D material demonstrates a nanosheet morphology with a thickness of ≈3 nm and lateral size of ≈0.45 μm. The statistical investigation on AFM and TEM profiles shows that the thickness distribution of the exfoliated 2D nanosheets with an average of 3.3 ± 0.5 nm and lateral dimensions 0.50 ± 0.35 μm (Figure 1c). Additional TEM and AFM images of the obtained 2D nanosheets are located in Figure SI‐1 and SI‐2, Supporting Information. Figure SI‐1, Supporting Information, presents the ultrathin 2D morphology for the obtained PdSO_4_ nanosheets based on the presented synthesis method, featuring a smooth surface, sharp edges, and clean corners. Figure 1d demonstrates the corresponding high‐resolution TEM (HRTEM) image with its selected area electron diffraction (SAED) pattern revealing the material is crystalline with *d*‐spacing of lattice fringes of 3.5 and 2.5 Å, which can be ascribed to the (110) and (200) lattice planes of monoclinic PdSO_4_ with the unit cell dimension ≈0.31 nm in the *c*‐direction (*c* = 61 910 Å), respectively.^[^ ^36^ ^]^ The unique anisotropic properties of PdS is possibly one of the main reasons to be exfoliated into layered‐like 2D structures.^[^ 37, 38 ^]^ In particular, the PdS bulk crystal exhibit a chain‐like structure, whereby Pd and S chains are held together by strong Pd—S covalent bonds but the adjacent chains are aggregating through weaker forces.^[^ 39, 40 ^]^ In addition, the neat form of PdS consists of lamellar assemblies, which can be disassembled into individual molecular nanosheets by dilution in polar organic solvents (e.g., DMF) and is capable of forming ultrathin layered‐like structures.^[^ 41, 42 ^]^ By applying the external force through the probe sonicator, the weak intrachain bonds can be broken within the DMF solvent and oxygen atoms will combine into the crystal structure of PdS during the process, eventually resulting in a 2D layered‐like structure of PdSO_4_.

**Figure 2:**
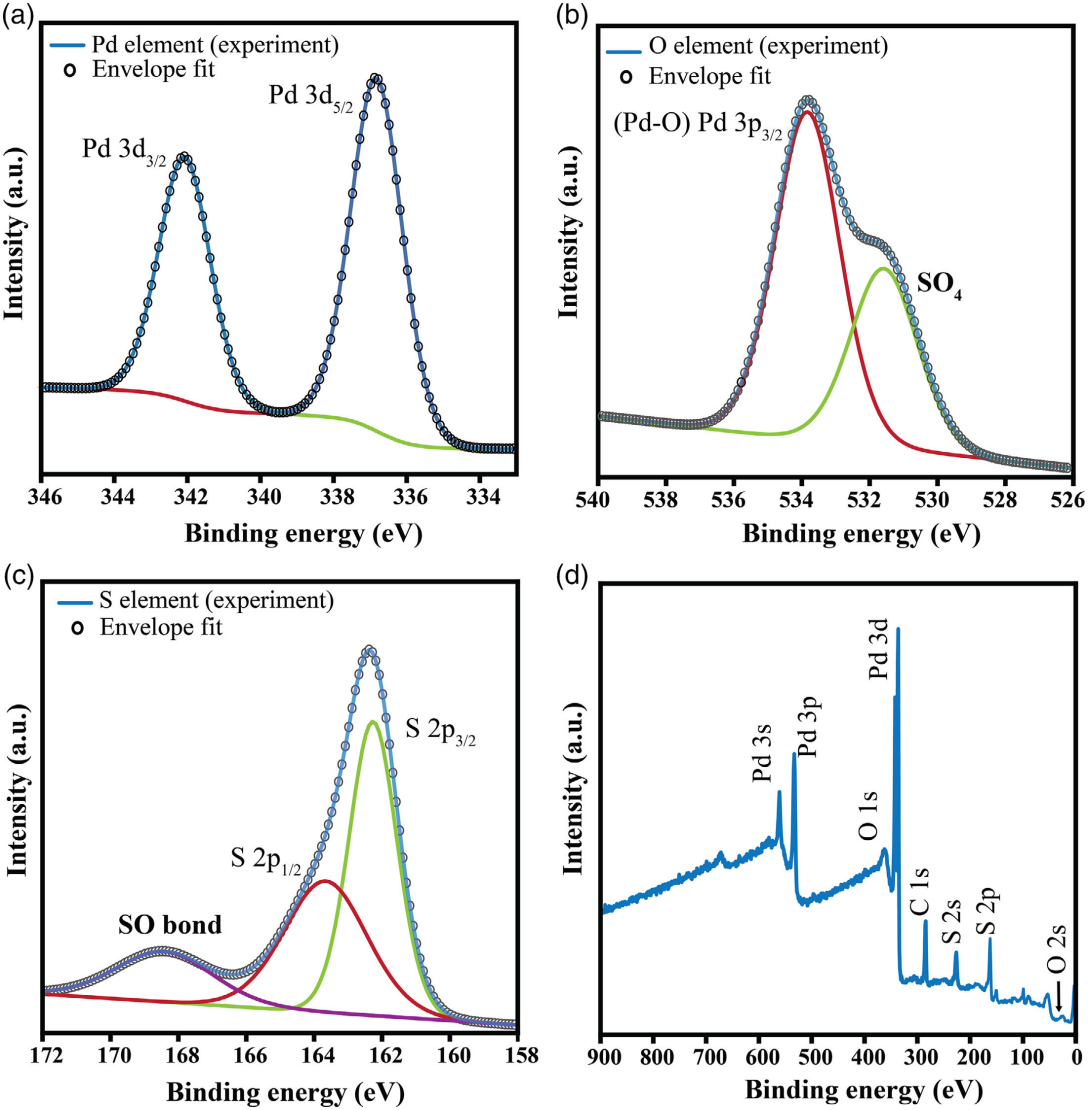
XPS spectra of 2D PdSO_4_ nanosheets for the regions of interest. High‐resolution spectrum for a) Pd 3*d*, b) S 2*p*, c) O 1*s*, and d) XPS survey spectrum.

The conversion of the materials to sulphate (SO_4_) was further investigated utilizing X‐ray photoelectron spectroscopy (XPS), energy dispersive spectroscopy (EDS), and X‐ray diffraction (XRD). XPS was employed to obtain the chemical bonding states of the produced 2D nanosheets. **Figure** **2**a shows the spectra of Pd 3*d* region of the synthesized 2D nanosheets. The characteristic main peaks located at binding energies of ≈336.6 and ≈341.8 eV correspond to Pd 3*d*_5/2_ and Pd 3*d*_3/2_, respectively.^[^ 43, 44, 45 ^]^ The high‐resolution O 1*s* spectrum shown in Figure 2b is fitted by two components. The peak at binding energy of ≈531.6 eV can be assigned to SO_4_ which is matching well with other sulphate groups with the value of 531.6 eV, 531.7 eV for aluminum sulphate Al_2_(SO_4_)_3_ and cadmium sulphate (CdSO_4_), respectively.^[^ 46, 47 ^]^ The peak centred at ≈533.73 eV corresponds well to Pd 3*p*_3/2_ spectra in the oxidation state of Pd which is in excellent agreement with the previous studies.^[^ 48, 49, 50 ^]^ The sulphur peak centered at a binding energy of 162.38 eV shown in Figure 2c is ﬁtted to identify the S 2*p* region, which was deconvoluted into two subpeaks located at binding energies of ≈162.30 and ≈163.65 eV correspond to 2*p*_3/2_ and 2*p*_1/2_, respectively.^[^ ^51^ ^]^ In addition, sulphate peak at binding energy ≈168.8 eV is observed due to the interaction of oxygen atoms into the Pd‐S atoms which can be assigned to the oxidized sulphur SO_4_. The chemical shift of the S element caused by the oxidation state that occurs during the exfoliation process is in good agreement with the previous reports.^[^ 52, 53, 54 ^]^ Figure 2d shows the XPS survey spectrum of the synthesized materials. Four elements were presented: Pd, S, O (from the sample), and C element located at 284.8 eV was used to calibrate the XPS spectral energies.^[^ 55, 56 ^]^ Furthermore, the chemical compositions of the obtained nanosheets are verified utilizing EDS elemental mapping on a single nanosheet shown in Figure SI‐3, Supporting Information. Figure SI‐3b,c, Supporting Information, confirms the homogenous distribution of Pd and S elements in the synthesized nanosheet, while Figure SI‐3d, Supporting Information, reveals the distribution of the O element across the nanosheet.

**Figure 3:**
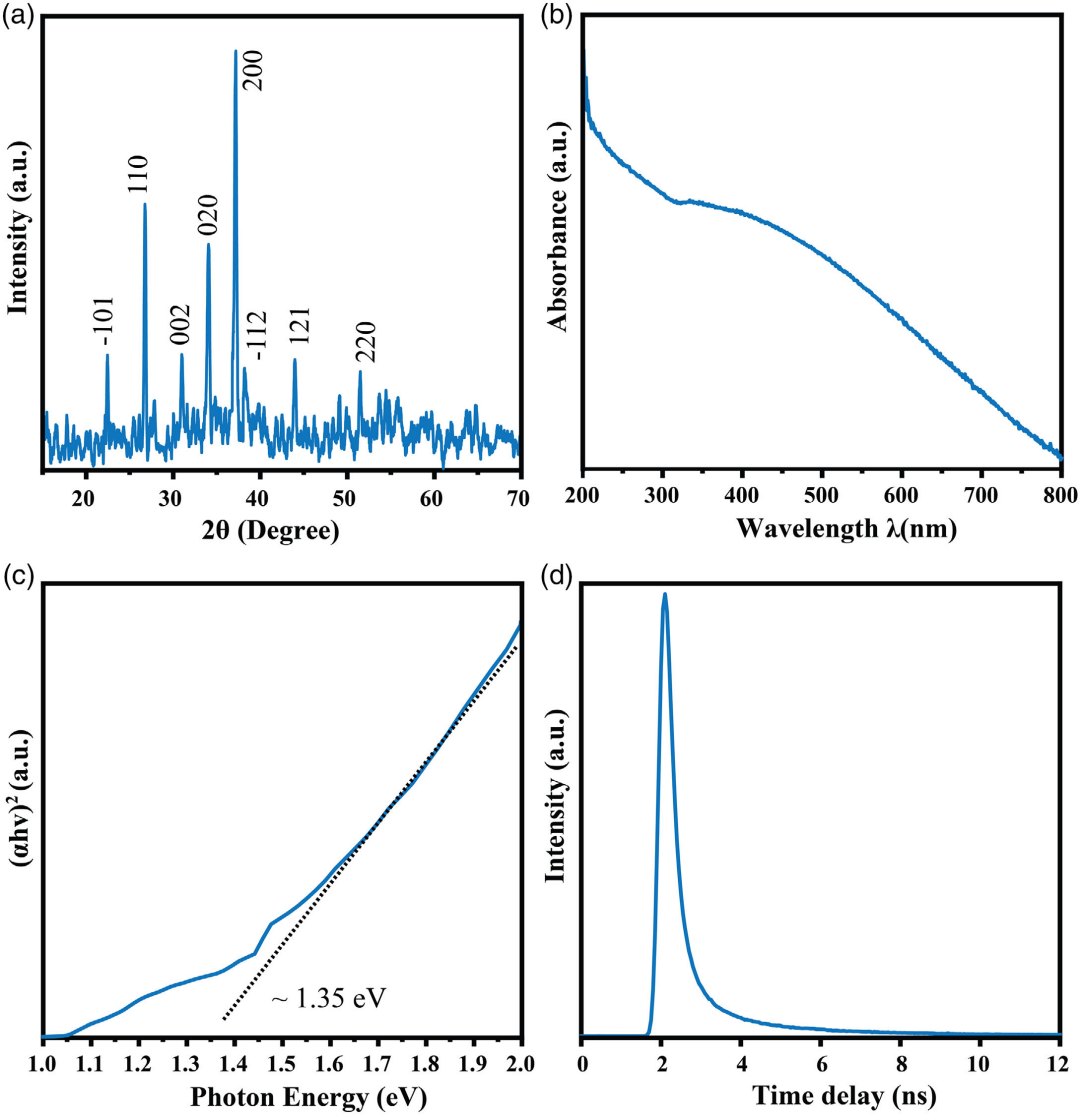
a) XRD spectrum. b) UV−vis absorption spectrum. c) The extracted Tauc plot with direct band gap ≈1.35 eV. d) PL decay curves of 2D PdSO_4_ nanosheets measured with laser excitation at 532 nm at room temperature.

XRD measurement was also utilized to explore the crystalline structure of 2D PdSO_4_ nanosheets. From **Figure** **3**a, the peaks at 22.4°, 26.76°, 31.04°, 34.10°, 37.19°, 38.29°, 43.99°, and 51.46° can be assigned to the (−101), (110), (002), (020), (200), (−112), (121), and (220) planes of monoclinic PdSO_4_, respectively.^[^ ^36^ ^]^ XRD and HRTEM results confirm the production of a monoclinic phase of PdSO_4_ with lattice spacings of *a* = 49 407 Å, *b* = 49 437 Å, and *c* = 61 910 Å. These findings indicate the successful transformation of the bulk material to palladium sulphate utilizing an inexpensive synthetic approach. The optical property of 2D PdSO_4_ nanosheets is shown in Figure 3b where the optical spectrum of synthesized 2D PdSO_4_ shows a broad absorption in the visible region ranging ≈400–620 nm.^[^ ^57^ ^]^ Thus, The synthesized 2D PdSO_4_ nanosheets are expected to be a promising material for optoelectronic devices, especially toward visible light.^[^ ^58^ ^]^ From the optical absorption, the synthesized 2D PdSO_4_ exhibits a narrow direct bandgap ≈1.35 eV which is determined using Tauc's analysis based on the difference between the band energy and the photon energy shown in Figure 3c.^[^ ^59^ ^]^ The average PL lifetime data of the 2D PdSO_4_ nanosheets is investigated and fitted based on the triple exponential decay model as in Equation (1) and calculated it following Equation (2)

(1) $$y=y_{0}+A_{1}e^{-(t-t_{0})/\tau_{1}}+A_{2}e^{-(t-t_{0})/\tau_{2}}+A_{3}e^{-(t-t_{0})/\tau_{3}}$$

(2) $$\tau_{\text{avg}}=\frac{A_{1}\tau_{1}^{2}+A_{2}\tau_{2}^{2}+A_{3}\tau_{3}^{2}}{A_{1}\tau_{1}+A_{2}\tau_{2}+A_{3}\tau_{3}}$$

where *A* is the amplitude, *τ* is the respective lifetime, and *t* is the time that has passed.^[^ ^60^ ^]^ The average excitons PL lifetime is shown in Figure 3d using excitation of a 532 nm laser. The extracted PL lifetime is 1.02 ns which is prolonged by one order comparing with other 2D materials such as Ga_2_S_3_, MoS_2_, WS_2_.^[^ 20, 61, 62 ^]^

**Figure 4:**
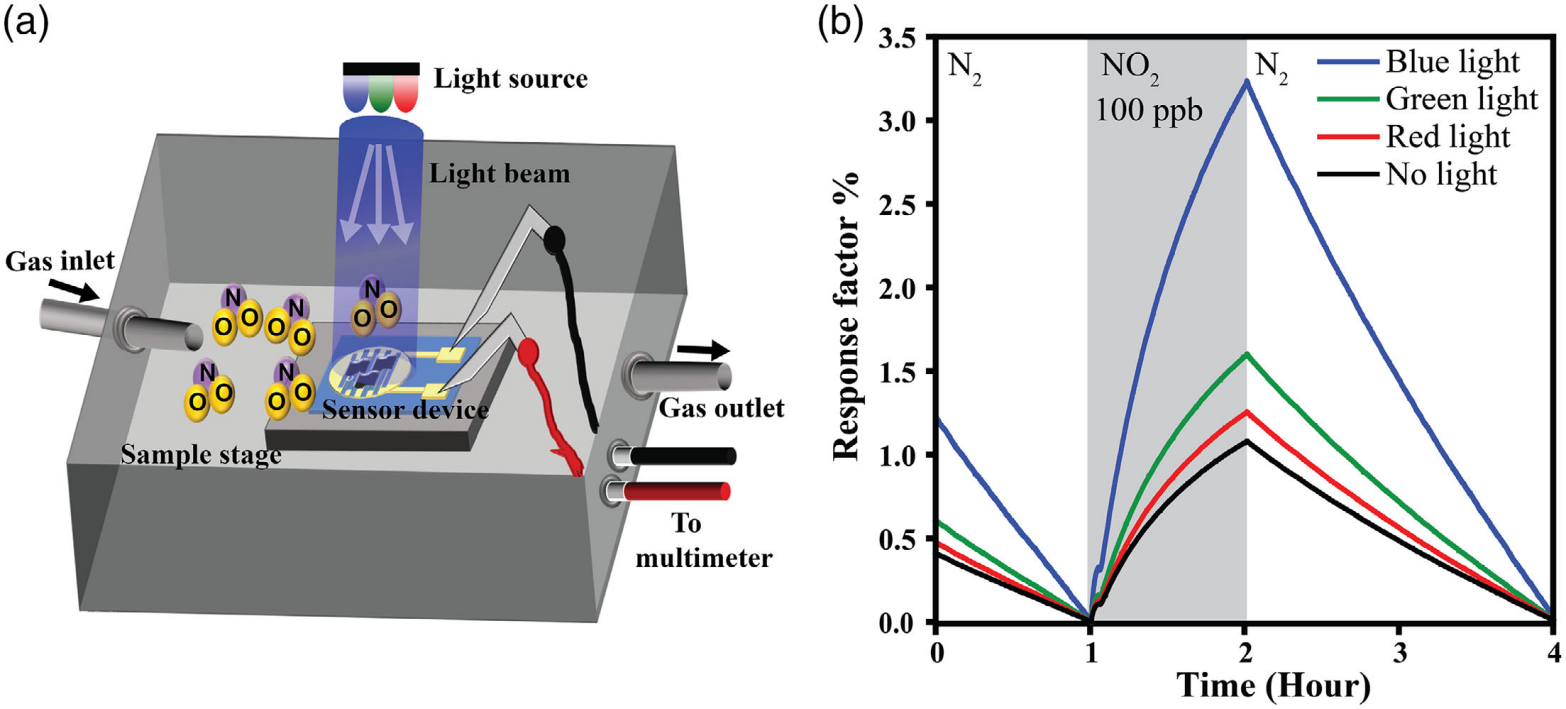
a) Schematic diagram of the gas testing chamber presenting the position of the light source and the 2D PdSO_4_ nanosheets which stack on the sensor electrode. b) Sensing performance of 2D PdSO_4_ nanosheets towards NO_2_ gas with concentration of 100 ppb in the dark condition and under red, green, and blue light illuminations at room temperature.

Gas sensors were fabricated by drop‐casting enough material of the synthesized 2D PdSO_4_ nanosheets on a precleaned 10 μm gap interdigital transducer (IDT) on an oxide‐coated silicon substrate to ensure the conductivity across the electrodes. 2D PdSO_4_ nanosheets will be laid between the top of electrode pairs, providing an efficient transport pathway of charge carriers.^[^ 63, 64 ^]^ The gas sensing performance was investigated using a fixed sensor testing chamber along with different wavelengths of 450, 520, and 620 nm at room temperature, as shown in **Figure** **4**a. The gas response factor was measured based on the electrical resistance of the sensor using (*R*_b_−*R*_g_)/*R*_b_ × 100%, where *R*_b_ represents the resistance of the device in the balancing gas (N_2_) and the *R*_g_ is defined as the resistance of the device under the analyte gas. The response and recovery time of the gas sensor are considered important parameters used for analysing a sensor, where the response time of the gas sensor is the time taken to attain 90% of the maximum saturation of the introduced gas, while the recovery time is considered as the time taken to achieve 10% of the sensor full recovery when the analyte gas is turned off. In the beginning, the optimal test condition was determined based on the visible light illuminations and dark conditions to analyze the 2D PdSO_4_ sensor behavior in a ppb level of NO_2_ gas at room temperate (Figure 4b). From Figure 3b,c, the 2D PdSO_4_ demonstrates a broad light absorption peak and a narrowed bandgap of 1.35 eV, revealing its outstanding photoexcitation behavior in the visible‐light spectrum. Moreover, as the material exhibited stronger light absorption properties on lower light wavelength (Figure 3b), the sensor was expected to perform better under a blue light source compared with others, which is consistent with our experimental results, as shown in Figure 4b, in which the response factor to 100 ppb of NO_2_ was ≈1.07 without illumination and ≈1.25, ≈1.60, ≈3.24 under the red, green, and blue illuminations, respectively. As a result, the intrinsic physisorption‐based gas sensing behaviors of the PdSO_4_ sensor are significantly improved by constantly applying an external light excitation. In particular, when exposed to the paramagnetic NO_2_ gas molecules acting as acceptors, surface dipoles are formed on 2D PdSO_4_ by attracting the electrons from the body, leaving the holes behind. Such a surface electron redistribution consequently causes the increase in electrical resistance in 2D PdSO_4_ with an n‐type semiconducting property, as the body hole concentration is enhanced. Furthermore, upon the light excitation, the photo‐excitons are generated and separated into electrons and holes efficiently in the presence of a relatively long exciton lifetime of 2D PdSO_4_, which are then diffused to the conduction band and valence band, respectively. Consequently, the photoinduced electrons in PdSO_4_ increase the available amounts of electrons for the interaction with the surface adsorbed NO_2_ gas molecules, enhancing the formation of surface dipoles between material and molecules.^[^ 65, 66, 67 ^]^ As a result, the sensor response factor upon the exposure of the same NO_2_ concentration is increased compared with that without light illumination. The blue light illumination condition (at ≈450 nm) exhibits the highest response factor of the sensor toward NO_2_ gas compared with those excited in green (≈520 nm) and red (≈620 nm) wavelengths, corresponding well to the optical absorption properties of 2D PdSO_4_ in the visible‐light regime, as presented in Figure 3b which is in excellent agreement with previous studies.^[^ 21, 68, 69, 70 ^]^

**Figure 5:**
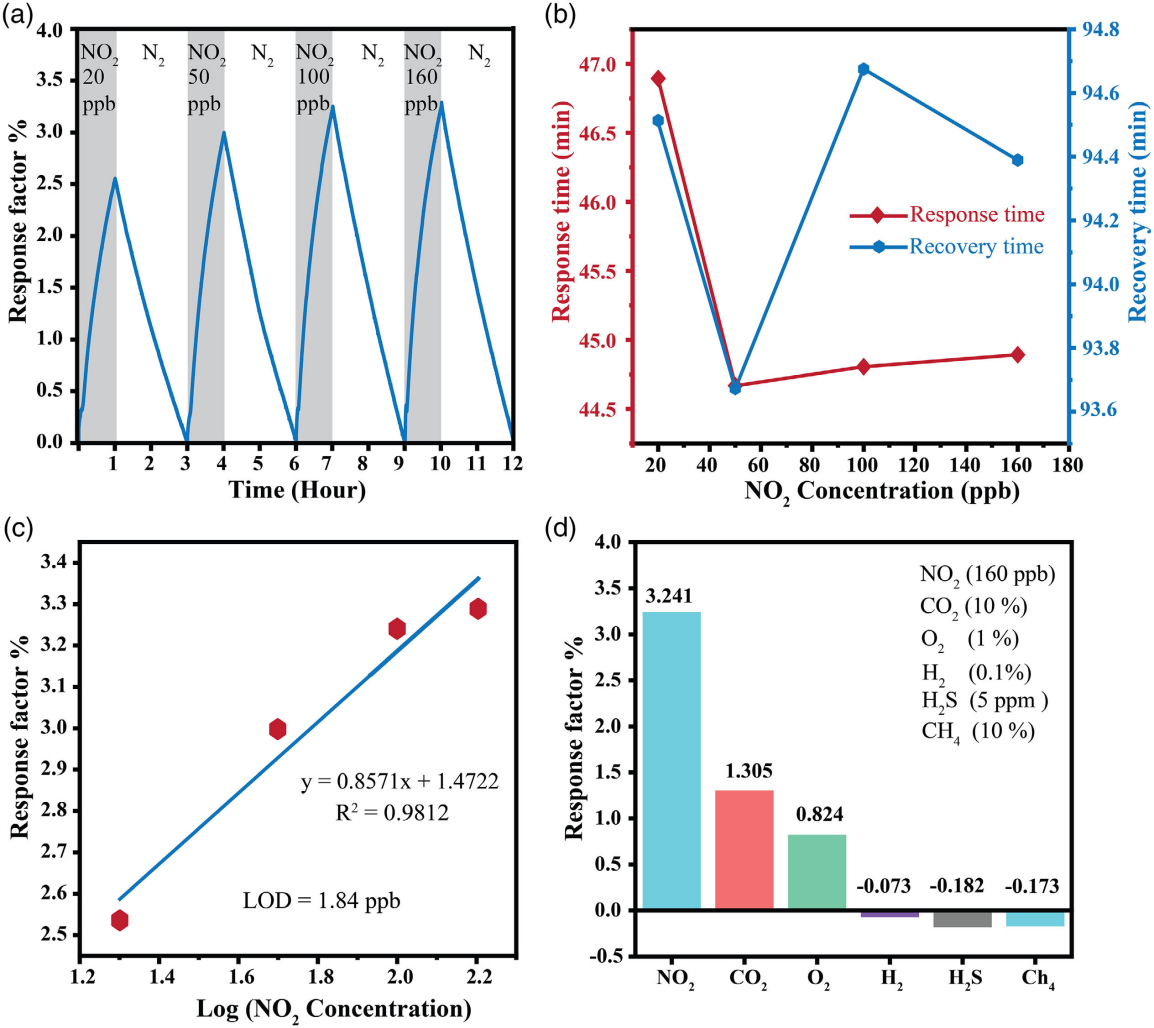
a) Sensing performance of 2D PdSO_4_ nanosheets under blue light illumination towards NO_2_ gas at concentrations ranging from 20 to 160 ppb at room temperature. b) The corresponding response and recovery time. c) The linear fitting between response values and Log (NO_2_ concentration) and limit of detection (LOD) calculation which the detection limits of 2D PdSO_4_ is estimated as 1.84 ppb. d) Detection selectivity of 2D PdSO_4_ sensor upon exposure to NO_2_ (160 ppb), CO_2_ (10%), O_2_ (1%), H_2_ (0.1%), H_2_S (5 ppm), and CH_4_ (10%).

As shown in **Figure** **5**a, the dynamic performance of the gas sensor was also assessed toward NO_2_ gas with concentrations ranging from 20 to 160 ppb in the balance of N_2_ gas, in which the highest response factor ≈3.28 for 160 ppb NO_2_ is demonstrated under the blue light illumination condition. Figure 5b shows the detailed information of the response and recovery times for the 2D PdSO_4_ as a function of NO_2_ concentrations extracted from Figure SI‐4, Supporting Information. The response time is notably decreased when increasing the NO_2_ concentration from 20 to 160 ppb and remains almost steady, possibly indicating the saturated surface of 2D PdSO_4_ for NO_2_ gas adsorption. However, the increase in the recovery time for NO_2_ concentrations beyond 50 ppb may be due to the small desorption rate of NO_2_ molecules from the interface of the 2D PdSO_4_ nanosheets.^[^ 71, 72 ^]^ According to Figure 5c, the limit of detection (LOD), calculated based on the standard deviation of the sensor response and the good linear slope between NO_2_ response values and the logarithmical values of NO_2_ concentrations, is found to be 1.84 ppb with a factor *R* ^2^ of 0.98. Such a small LOD is in the top range for optoelectronic gas sensors operated at room temperature (Table SI‐2, Supporting Information).^[^ ^73^ ^]^ Under the same testing condition, other commonly seen industrial gases such as O_2_ (1%), CO_2_ (10%), H_2_ (0.1%), H_2_S (5 ppm), and CH_4_ (10%) show response factors of ≈0.824, ≈1.305, ≈−0.073, ≈−0.182, and ≈−0.173, respectively (Figure 5d). Figure SI‐6, Supporting Information, shows the corresponding sensing response curves. In comparison with NO_2_ at a relatively small concentration level (160 ppb), the gas response factor for NO_2_ is at least three times enhanced. Moreover, the PdSO_4_‐based sensor was tested against the environments with 60 ppb of NO_2_ gas at 30% and 50% relative humidity (RH). As shown in Figure SI‐7, Supporting Information, the response factor was dropped from 3.19 at the dry condition to 3.1 and 2.9 for 30% RH and 50% RH, respectively, possibly due to the adsorption of water vapor as electron donors upon the PdSO_4_ surface.^[^ 74, 75 ^]^ However, such an influence of humidity on the material is insignificant considering its negligible degradation of sensing performance.

## 3 Conclusion

We successfully synthesized 2D PdSO_4_ nanosheets from microsized PdS bulk powder through the facile liquid‐phase exfoliation method. During the mechanical agitation under the high power of the probe sonicator, the bulk PdS was exfoliated and thinned down to the 2D domain, while the active oxygen species in the solvent reacted with PdS and transformed into PdSO_4_ evidenced by the detailed investigations of the XPS, EDS mapping, and XRD. The produced nanomaterials featured translucent 2D nanosheets with average lateral dimensions 0.50 ± 0.35 μm and thickness 3.3 ± 0.5 nm. It was also found that the 2D PdSO_4_ nanosheets exhibited an optical absorption across the visible region with a narrow bandgap of 1.35 eV. Furthermore, we examined the possibility of implementing 2D PdSO_4_ for room temperature optoelectronic sensing applications given its strong excitonic interaction evidenced by the prolonged exciton lifetime. The LEDs with the wavelength regions of blue, green, and red, were used as the excitation source and paramagnetic NO_2_ gas was selected as a representative gaseous analyte. The positive response of the sensor based on 2D PdSO_4_ nanosheets toward the NO_2_ gas as acceptors indicated the n‐type semiconducting feature. The sensor demonstrated the highest response under the blue light illumination over other visible‐light regions owing to the optical absorption property of 2D PdSO_4_. Room temperature reversible sensing performances were also achieved with a response factor of ≈3.28% at NO_2_ concentration of 160 ppb, a low LOD of ≈1.84 ppb, and a minimum three times enhancement on the response factor compared with other commonly seen industrial gases with meaningful concentrations. This work demonstrates the possibility of producing 2D noble metal sulphide compounds given their nonlayered bulk crystal structure and the strong potentials in the implementation of this emerging group of 2D materials for developing high‐performance optoelectronic devices and sensors.

## 4 Experimental Section

### Synthesis of 2D PdSO_4_ Nanosheets

The material and the chemicals solvents were used directly without any further purification. 200 mg of microsized PdS bulk powder (99.5% metals basis, Alfa Aesar) was mixed with 300 μL of *N*,*N*‐dimethylformamide DMF (99.8%, Sigma‐Aldrich) and mechanically ground using a mortar and pestle for 30 min. The ground mixture was dispersed in 25 mL of DMF solvent for additional breaking up with a probe sonicator (Ultrasonic Processor GEX500) for 2 h at the power of 100 W. After that, the obtained solution was then extracted by centrifugation at 3500 revolutions per minute (RPM) for 30 min. Then, the collected supernatant having 2D PdSO_4_ nanosheets was further centrifuged at the speed of 12 000 RPM for 25 min and repeated the washing step two times using ethanol to separate the exfoliated nanosheets from the DMF solvent. The exfoliated 2D nanosheets was kept in ethanol solvent to further investigation.

### Characterizations

The lateral dimensions and morphologies of the synthesized 2D nanosheets were characterized using TEM on a JEOL 1010 TEM with accelerating voltages of 100 kV. HRTEM on a JEOL 2100F with accelerating voltages 200 kV was utilized to study the crystal structures of the obtained materials. The lateral dimensions and thicknesses of 2D PdSO_4_ nanosheets were measured using an AFM (Bruker Dimension Icon) in the ScanAsyst air mode with a ScanAsyst tip. XPS analysis was performed on a Thermo Scientific K‐Alpha instrument spectrometer with an Al Kα radiation source. The XRD measurements of the synthesized materials were conducted using a Bruker D4 Endeavor with Cu Kα radiation of 1.5406 Å. The UV–vis–NIR absorption spectra were collected using an Agilent Cary 60 Spectrophotometer. Scanning electron microscopy (SEM, FEIQuanta 200) and Oxford *x*‐MaxN 20 energy‐dispersive X‐ray spectrometer (EDS) were utilized to perform the elemental mapping analysis of the exfoliated samples. A home‐built confocal microscope was used to perform lifetime measurements for the synthesized materials. A supercontinuum laser (NKT Photonics, Fianium WhiteLase) set at a wavelength of 532 nm (25 nm bandwidth) and repetition rate of 2 MHz used as an excitation source. Excitation and detection were fiber‐coupled, the beam passed through an ND filter wheel to adjust the laser power manually. A 532 nm dichroic mirror reflected the beam onto two mirrors to focus the beam onto the sample via a dry objective (100×, 0.9 NA). The emissions were collected back using the same objective and transmitted through the dichroic mirror and fiber‐coupled multimode. The fiber‐connected directly to APD detectors (SPCM‐AQRH‐14, Excelitas) were employed for imaging and analysing with a correlator card (Picoquant, TimeHarp 260) for time‐resolved measurements.

### Sensor Fabrication and Measurement

A well‐designed gas transducer was fabricated upon a silicon dioxide substrate with 200 pairs of 10 μm wide interdigitated electrodes (IDE) gold electrodes. 15 μL of suspension containing 8 mg mL^−1^ of 2D PdSO_4_ nanosheets was drop‐casted at 35 °C on the prepared transducer. The gas sensor experiments were conducted in a sealed gas chamber at room temperature. The change in resistance of the sensor was measured utilizing an Agilent 34401A digital multimeter. During the measurements, an excitation light source (Cree XLamp XM‐L) with distinct wavelengths of 450, 520, and 620 nm was applied to the sensors through a quartz window on the top of the chamber. The gas input stream was supplied in via a programmable multichannel mass flow control (MKS 1479A, USA) at a stable flow rate of 2000 standard cubic centimeter per minute (sccm). Different concentrations of NO_2_ gas were scrutinized. Additionally, the selectivity testing was investigated based on other common gases such as carbon dioxide (CO_2_), O_2_ (1%), hydrogen (H_2_), hydrogen sulphide (H_2_S), and methane (CH_4_).

## Conflict of Interest

The authors declare no conflict of interest.

## Supporting information

Supplementary Material

## Data Availability

Research data are not shared.
