# Supplementary material for: 2D Palladium Sulphate for Visible‐Light‐Driven Optoelectronic Reversible Gas Sensing at Room Temperature
Source: Small Sci. 2021 Dec 8;2(3):2100097. doi: 10.1002/smsc.202100097 (PMC11935894; doi:10.1002/smsc.202100097)
Supplement: Supplementary file 1 — Supplementary Material [file SMSC-2-2100097-s001.pdf]

Supporting Information

Two-dimensional palladium sulphate for visible-light-driven optoelectronic reversible gas sensing at room temperature

Turki Alkathiri, Kai Xu, Bao Yue Zhang, Muhammad Waqas Khan, Azmira Jannat, Nitu Syed, A.F.M. Almutairi, Nam Ha, Manal M. Y. A. Alsaif, Naresha Pillai, Zhong Li,* Torben Daeneke, and Jian Zhen Ou**

T. Alkathiri, Dr. K. Xu, Dr. B. Zhang, M. Khan, Dr. A. Jannat, Dr. N. Syed, A. Almutairi, N. Ha, Dr. M. Alsaif, Dr. N. Pillai, Dr. T. Daeneke, Prof. J. Z. Ou,

School of Engineering

RMIT University, Melbourne, 3001, Australia

E-mail: jianzhen.ou@rmit.edu.au; kai.xu@rmit.edu.au; nuaalizhong@163.com

T. Alkathiri

School of Engineering

Albaha University, Albaha 65779, Saudi Arabia

Dr. M. Alsaif

Department of Electrical Engineering

Kuwait University, Safat 13060, Kuwait

Prof. Z. Li,

Key Laboratory of Advanced Technologies of Materials, School of Materials Science and Engineering

Southwest Jiaotong University, Chengdu 610031, China

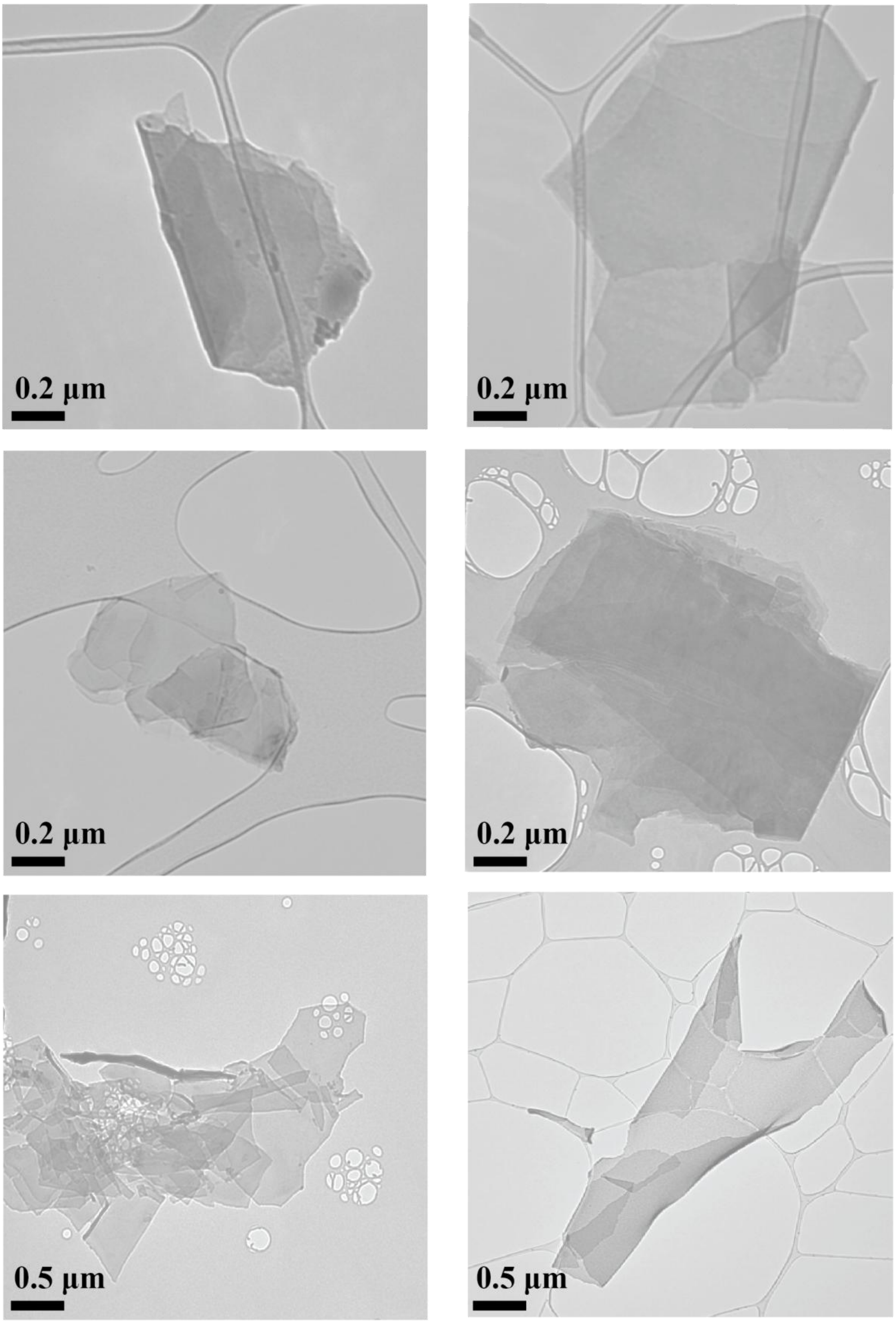

Figure SI-1. Typical TEM images of prepared 2D $PdSO_4$ nanosheets from multiple spots.

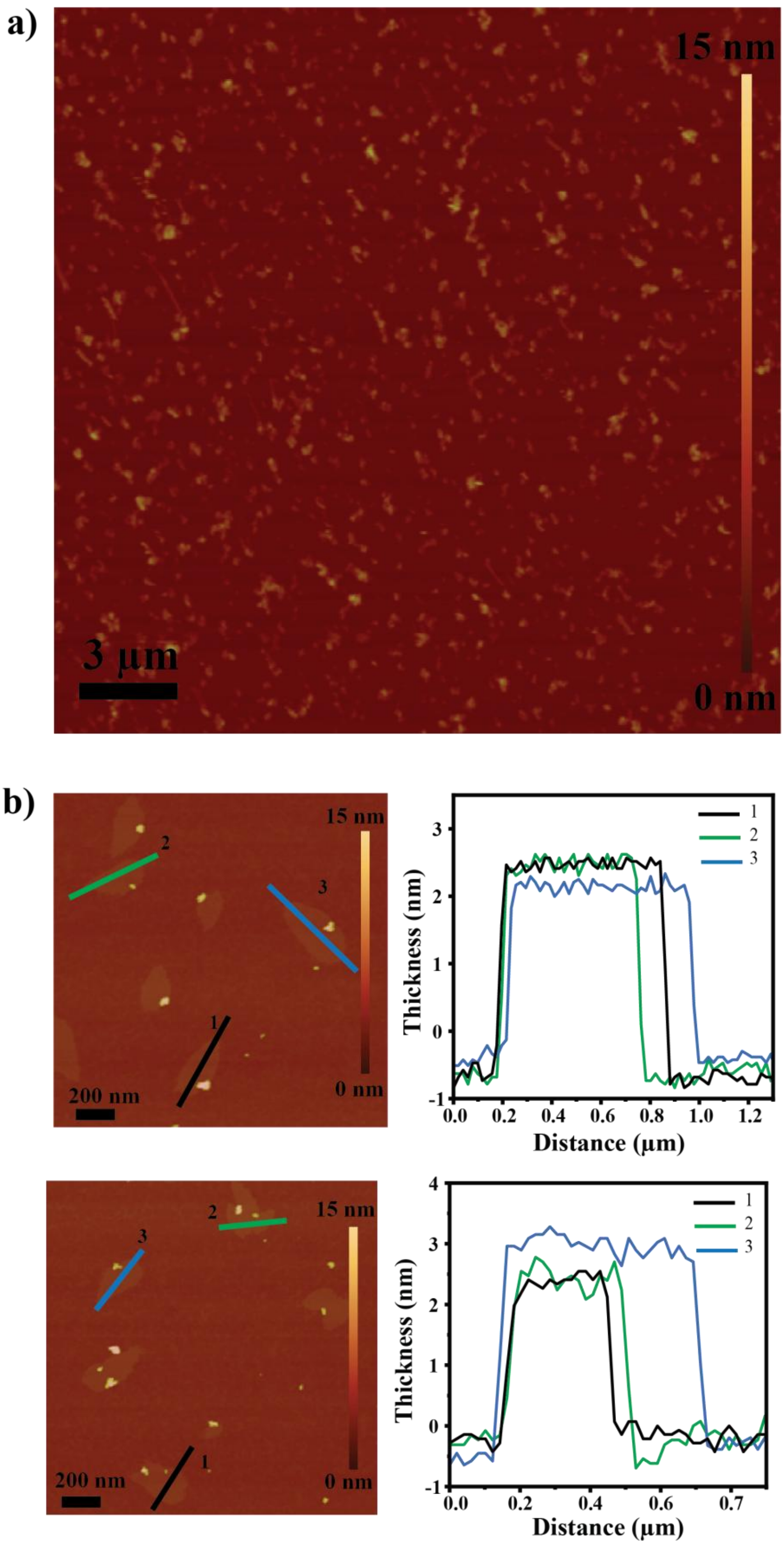

Figure SI-2. a) A large-scale AFM image of the exfoliated 2D PdSO4 nanosheets (20 µm by 20 µm).

b) Typical AFM images of the obtained nanosheets and corresponding thickness profiles.

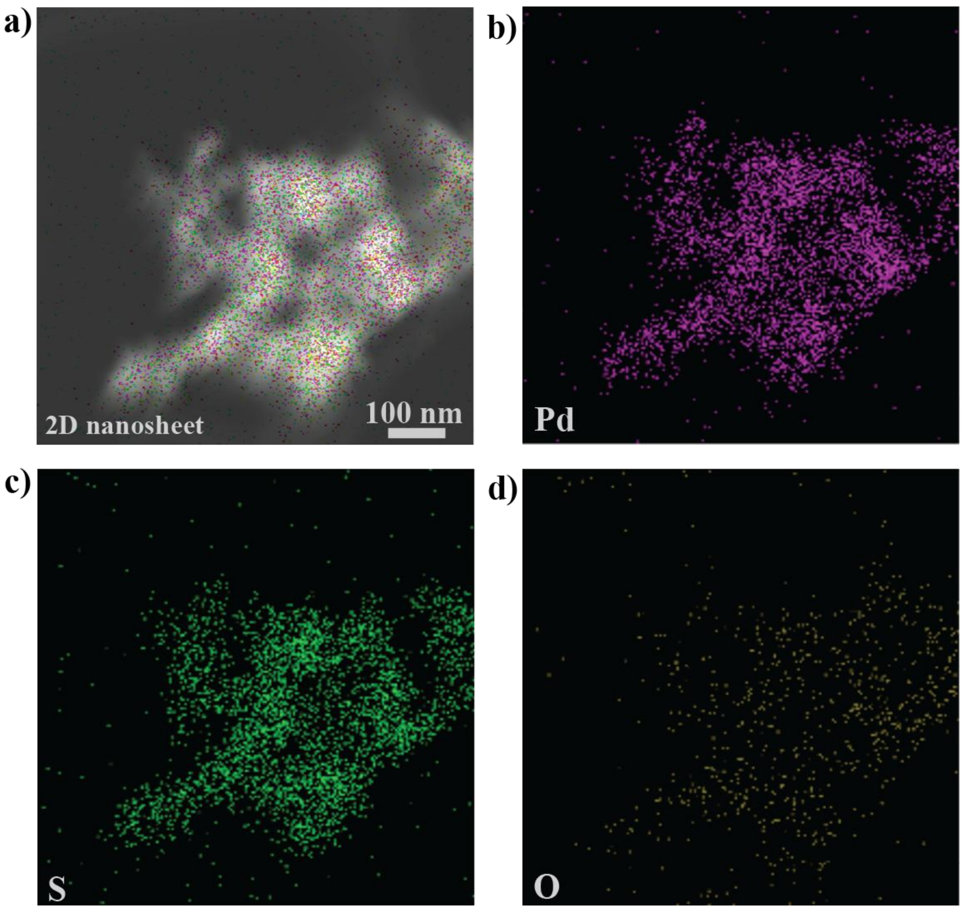

Figure SI-3. SEM-EDS elemental mapping: (a) Elemental mapping image of a selected area on the sample. (b) Pd mapping. (c) S mapping. (d) O mapping.

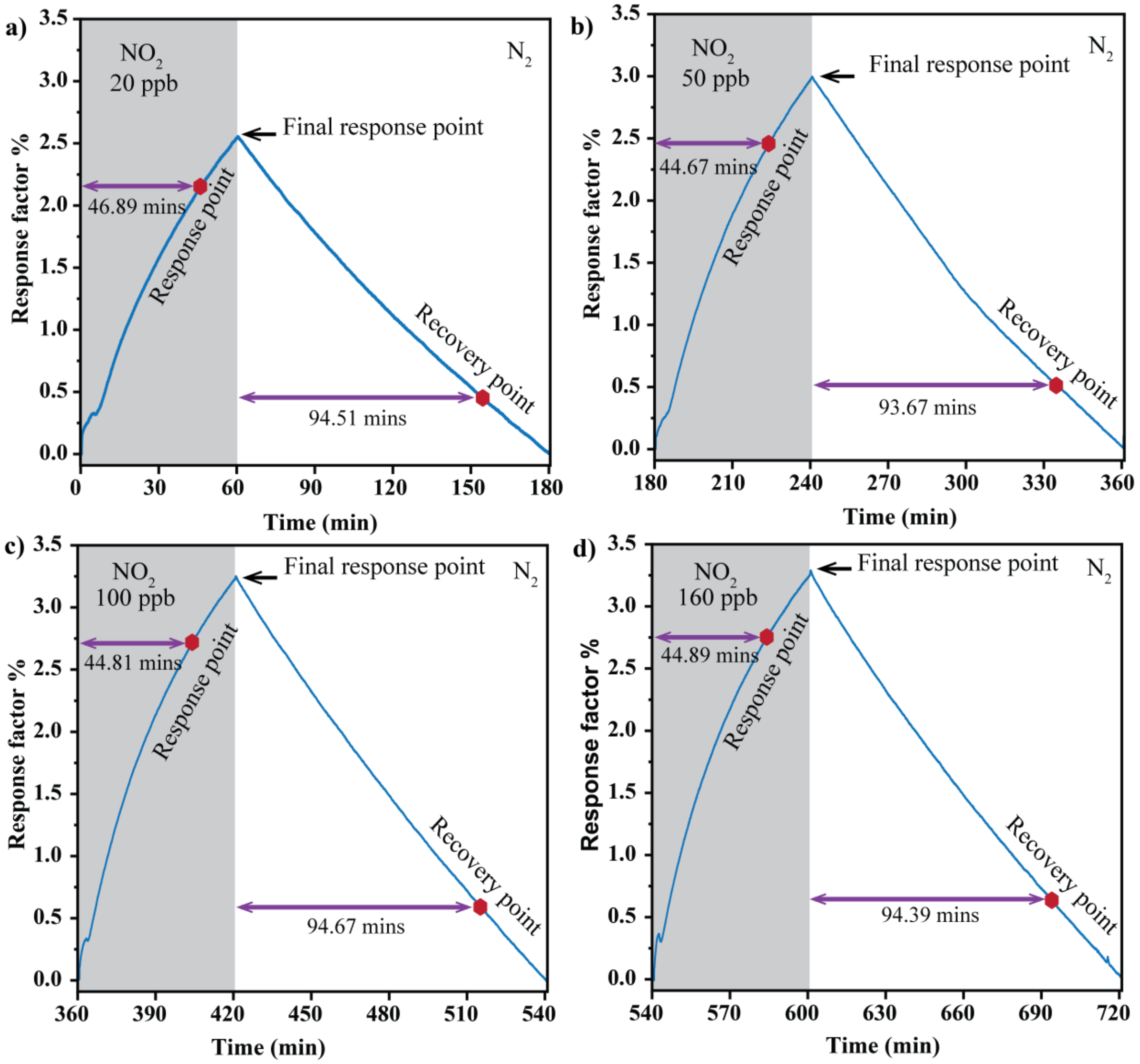

Figure SI-4. The response and recovery measurements of 2D $PdSO_4$ sensor toward NO_2 concentration of (a) 20, (b) 50, (c) 100, and (d) 160 ppb.

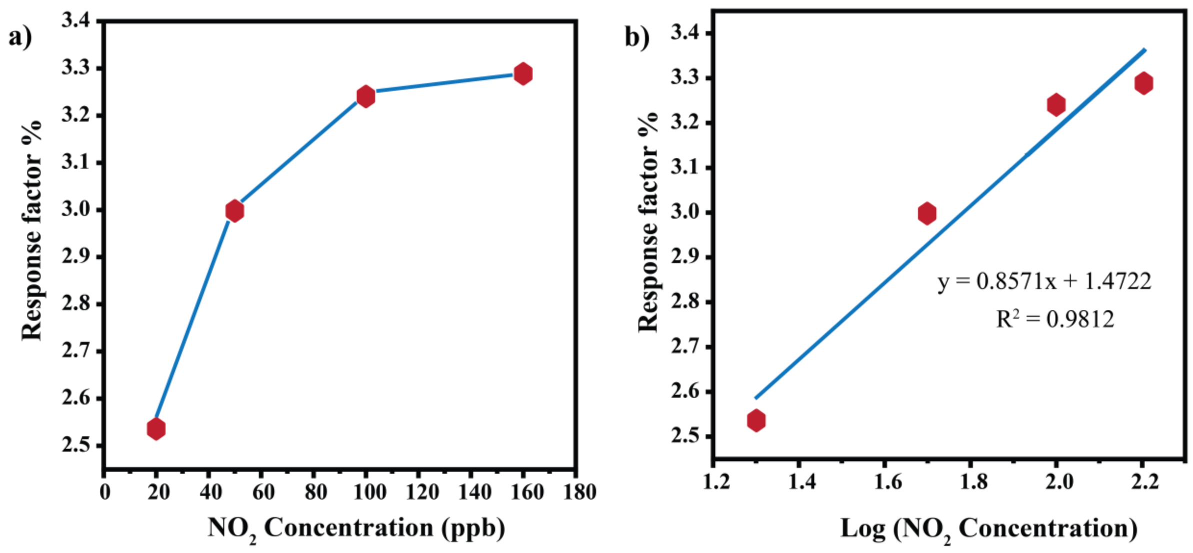

Figure SI-5. a) Response factor of 2D $PdSO_4$ based sensor with the increase of NO_2 concentrations. b) The linear fitting of the sensor response factors toward the logarithm scale of NO_2 concentrations of 20, 50, 100, and 160 ppb.

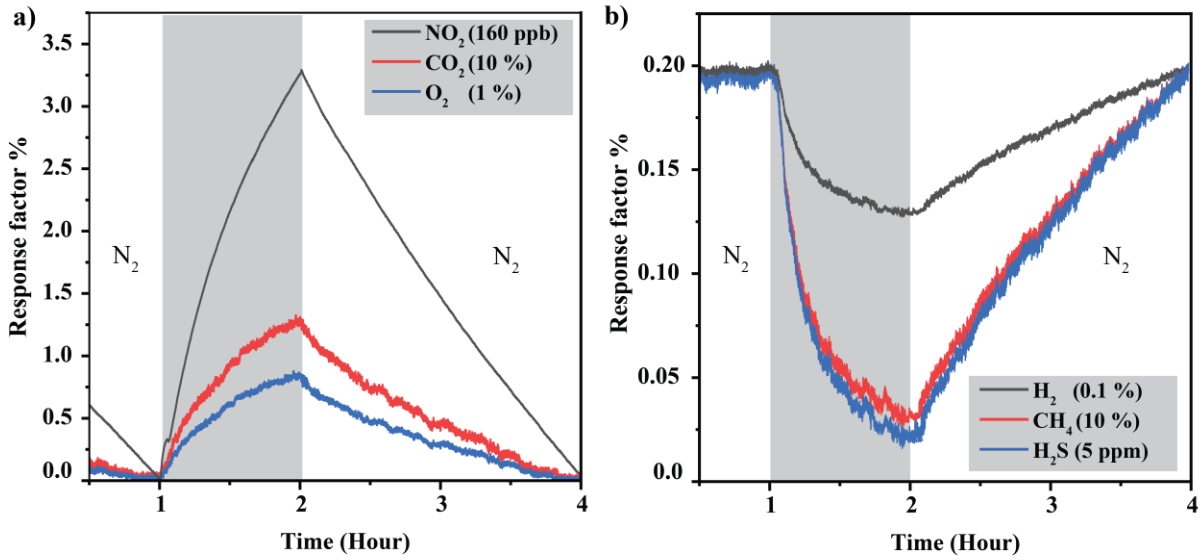

Figure SI-6. a) Response curves of 2D $PdSO_4$ based sensor toward various oxidizing gases: NO_2 (160 ppb), CO_2 (10%), and O_2 (1%). b) Response curves of 2D $PdSO_4$ based sensor toward various reduction gases: H_2 (0.1 %), CH_4 (10%), and H_2S (5 ppm).

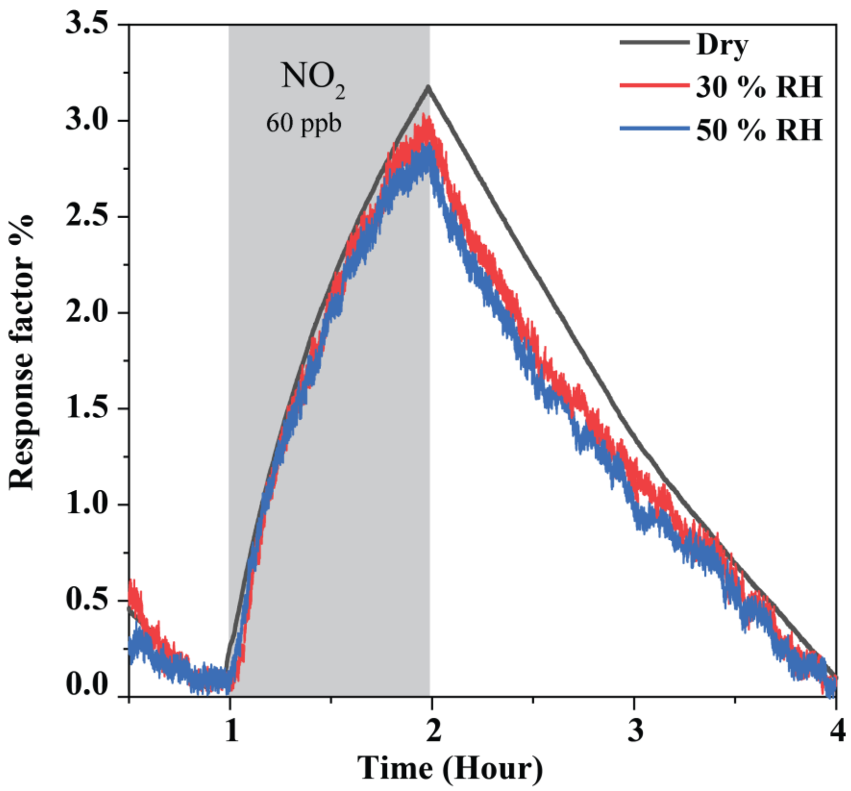

Figure SI-7. The response curves of 2D $PdSO_4$ based sensor toward 60 ppb NO_2 in the dry, 30% RH, and 50% RH conditions.

Table SI-1. The PL lifetime fitting result 2D $PdSO_4$ nanosheets after using the triple exponential decay model.

	A_1	t_1	A_2	t_2	A_3	t_3
**2D $PdSO_4$**	42537.33158	0.30712	34909.02317	0.30714	6043.27413	2.2745
	Average lifetime (ns)= 1.028					

Table SI-2. Limit of detection of NO_2 toward 2D materials under visible light illuminations at room temperature.

**Materials**	**Light source (nm)**	**Smallest tested NO_2 Concentration (ppm)**	**LOD (ppb)**	**Ref**
ZnO-Ag nanoparticles	470	0.5	500	[1]
ZnO/g-C_3N_4	460	1	38	[2]
ZnO/PbS	850	1	26	[3]
rGO/MoS_2	405	50	53	[4]
$PdSO_4$	450	0.02	1.84	This work

References.

1. Q. Zhang, G. Xie, M. Xu, Y. Su, H. Tai, H. Du, Y. Jiang, *Sens. Actuators, B* **2018**, *259*, 269.
2. H. Wang, J. Bai, M. Dai, K. Liu, Y. Liu, L. Zhou, F. Liu, F. Liu, Y. Gao, X. Yan, *Sens. Actuators, B* **2020**, *304*, 127287.
3. R. Chen, J. Wang, Y. Xia, L. Xiang, *Sens. Actuators, B* **2018**, *255*, 2538.
4. Y. Niu, R. Wang, W. Jiao, G. Ding, L. Hao, F. Yang, X. He, *Carbon* **2015**, *95*, 34.
